# Submicroscopic chromosome imbalance in patients with developmental delay and/or dysmorphism referred specifically for Fragile X testing and karyotype analysis

**DOI:** 10.1186/1755-8166-1-2

**Published:** 2008-03-26

**Authors:** Joo Wook Ahn, Kathy Mann, Zoe Docherty, Caroline Mackie Ogilvie

**Affiliations:** 1 Cytogenetics Department, Guy's and St Thomas' NHS Foundation Trust, London, UK

## Abstract

**Background:**

Microdeletion syndromes are generally identified because they usually give rise to specific phenotypic features; many of these deletions are mediated by duplicons or LCRs. The phenotypes associated with subtelomeric deletions are also becoming recognised. However, reciprocal duplication events at these loci are less easily recognised and identified, as they may give rise to milder phenotypic features, and the individuals carrying them may not therefore be referred for appropriate testing. 403 patients with developmental delay and/or dysmorphism, referred to our Genetics Centre for karyotyping and Fragile X expansion testing, were assessed for chromosome imbalance by Multiplex Ligation-dependent Probe Amplification (MLPA). Two MLPA kits were used, one containing probes for the subtelomere regions, and one containing probes for common microdeletion loci. 321 patients were tested with both kits, 75 with the subtelomere kit alone, and 7 with the microdeletion kit alone.

**Results:**

32 patients had abnormal results; the overall abnormality detection rate was 2.5% for karyotype analysis and 7.2% for MLPA testing; 5.5% of subtelomere tests and 2.1% of microdeletion tests gave abnormal results. Of the abnormal MLPA results, 5 were in cases with cytogenetically visible abnormalities; of the remaining, submicroscopic, changes, 3 results were established as de novo and 8 were inherited; parental samples were not available for the remaining cases. None of the patients was found to have a Fragile X expansion.

**Conclusion:**

Karyotype analysis in combination with MLPA assays for subtelomeres and microdeletion loci may be recommended for this patient group.

## Background

Microdeletion syndromes are generally caused by recurrent, duplicon- or LCR-mediated, contiguous gene deletions, which are identified because they usually give rise to specific phenotypic features. Similarly, the phenotypes associated with subtelomeric deletions are becoming recognised [1]. However, the reciprocal duplication events at these loci may give rise to milder phenotypic features, and the individuals carrying these duplications may not be referred for appropriate testing.

Multiplex Ligation-dependent Probe Amplification (MLPA) [2] is a molecular method for assessing copy number at multiple loci in the genome, and has advantages over Fluorescence In Situ Hybridization (FISH) in terms of throughput of testing, multiplexing of loci, and unequivocal identification of duplications as well as deletions. Previous reports of the use of MLPA for screening individuals with idiopathic mental retardation and/or dysmorphism include cohorts of 51, 75, 210 and 455 tested for subtelomere imbalance [3-6], 258 patients tested for imbalance at microdeletion loci [7], and 58 patients tested for both subtelomere and microdeletion loci [8]. At our Centre, such patients are routinely referred for karyotype analysis and Fragile X testing.

We have carried out testing for imbalance at microdeletion sites and in the subtelomere regions of chromosomes in a cohort of 403 patients referred with developmental delay and/or dysmorphism, with the aim of assessing the prevalence of imbalance at these loci in patients in this referral group, and to provide diagnoses for those who had normal karyotypes and no Fragile X expansion mutations. This cohort did not include patients whose referrals included requests for specific microdeletion tests.

## Results

A total of 403 patients was tested, 321 with subtelomere and microdeletion kits, 75 with the subtelomere kit(s) alone, and 7 with the microdeletion kit alone. 32 patients had abnormal results: 10 patients had cytogenetically visible chromosome abnormalities (detailed in Table 1). Of these, one had an inherited balanced rearrangement (#8), one an apparently balanced inversion (#5), one carried Yq heterochromatin on the chromosome 21 short arm (#6), one had a structurally abnormal Y chromosome (#1), and two had numerical sex chromosome abnormalities (#9 and #10). None of these findings is considered likely to be associated with the referral indications of developmental delay and dysmorphism, although gene disruption at the breakpoints of case #5 cannot be excluded. Clinically significant unbalanced karyotypes, representing 1.0% (4/403) of the patient group, comprised a derivative chromosome 10 (#3) and an abnormal chromosome 20 (#4), both also identified by MLPA, and two interstitial deletions (#2 and #7).

**Table 1:** Karyotypes in patients found to have abnormalities on G-banded chromosome analysis

Patient	Age	Karyotype	MLPA MD	MLPA ST
1	5	46,X,der(Y)inv(Y)(p11q11.2)Yqs pat.ish der(Y)(DXYS130 st,DXYS224 st)	NAD	NAD
2	6	46,XX,del(12)(q21.1q21.2)	NAD	NAD
3	5	46,XX,der(10)t(7;10)(q36.1;q26.3).ish der(10)(D10S2490-,D7S427+)	NAD	del 10q
4	1	46,XX,der(20)dup(20)(p13p11.2)del(20)(p13)dn.ish der(20)(D20S1156-,wcp20+,pcp20p+, pcp20q+,RH44234+)	Alagille duplication	del 20p
5	5	46,XX,inv(18)(q12.2q23)	NAD	NAD
6	1	46,XY,?v21pss	NAD	dup XYq
7	1	46,XY,del(7)(p1?4.2p1?5.1)	NAD	NAD
8	3	46,XY,inv(1)(p36.22p36.33)mat.ish inv(1)(CEB108 st,D1Z2 mv)	NAD	NAD
9	1	47,XXY	NAD	XXY
10	2	48,XXYY	NAD	dup XYp, dup XYq

NAD – No abnormality detected

Table 2 shows the abnormalities detected by the microdeletion MLPA kit. Although parents were only available for inheritance studies for patients #4 and #11, it is very likely that the other abnormalities in this group also arose de novo, and were associated with the clinical phenotype of these patients. The finding in patient #4 was consistent with the abnormal karyotype, whilst patient #16, with a single probe duplication in the DiGeorge critical region, also carried a deletion of chromosome 8 short arm material (See Table 3).

**Table 2:** Details of imbalances found using the MLPA microdeletion kit

Patient	Age	MLPA MD	Follow-up
4	1	Alagille duplication	abnormal karyotype, de novo
11	1	Prader-Willi deletion	confirmed by FISH, de novo
12	11	Williams deletion	confirmed by FISH
13	3	Williams deletion	confirmed by FISH
14	9	DiGeorge duplication	confirmed by FISH
15	5	DiGeorge partial duplication	confirmed by FISH
16	3	DiGeorge duplication	

**Table 3:** Details of abnormalities found using the MLPA subtelomere kit

Patient	Age	MLPA ST	Follow-up
24	7	del 2q (1), dup 22q (2)	confirmed by FISH
19	3	del 3p (1)	
27	11	del 3p (2)	carried by maternal aunt
17	2	del 4q (1)	
26	22	dup 5q (2)	
30	3	dup 5q (2)	
29	6	dup 6p (2)	carried by affected sister
28	3	dup 8p (1)	confirmed by FISH
16	3	del 8p (2)	confirmed by FISH
31	35	del 8p (1)	
18	9	dup 9p (1), dup XYp (2), del XYq (2)	9pdup maternal, abnormal Y confirmed by FISH
21	4	dup 9p (1)	maternal
25	2	dup 9p (1)	paternal
20	7	dup 16p (1)	maternal
32	1	dup 21q (1)	
22	2	dup XYp (2)	paternal
23	2	dup XYq (2)	confirmed by FISH
3	5	dup 7q (2), del 10q (2)	abnormal karyotype
6	1	dup XYp, dup XYq	abnormal karyotype
10	2	dup XYp, dup XYq	abnormal karyotype
4	1	del 20p	abnormal karyotype
9	1	XXY	abnormal karyotype

Numbers in parentheses indicate the number of probes showing abnormal copy number. "confirmed by FISH" indicates that a probe for the relevant subtelomere region showed a concordant result.

Table 3 shows the subtelomere MLPA abnormalities detected. Five of these confirmed abnormal karyotypes (#3, #4, #6, #9 and #10). There were 3 inherited duplications of 9p (#21, #20 and #25), and three imbalances of 8p (#16, #28, #31), two of which were confirmed by FISH. Patient #24 was found to carry a derivative chromosome 2 from a 2q;22q translocation. Retrospective examination of G-banded chromosomes from this case showed that the translocation was submicroscopic. Patient #18 carried two abnormalities: an inherited duplication of material from the subtelomere region of the chromosome 9 short arm and in addition a structurally abnormal Y chromosome, not originally identified by G-banded chromosome analysis. Nine of the subtelomere anomalies involved only a single MLPA probe, and therefore required confirmation; of these, 4 were confirmed in other family members, one was confirmed by FISH, and 4 remain unconfirmed. Parental or other family samples were only available for 7 of the 17 cases with submicroscopic abnormalities in this group, all of which showed inheritance of the anomalies, although in some cases no information was available on the phenotype of the carrier parent.

None of the cases in the patient cohort carried Fragile X expansion mutations. The overall abnormality detection rate was therefore 0% (0 cases) for Fragile X testing, 2.5% (10 cases) for karyotype analysis and 7.2% (29 cases) for MLPA testing; 5.5% (22 cases) of subtelomere tests and 2.1% (7 cases) of microdeletion tests gave abnormal results.

## Discussion

We have previously assessed and validated the commercially available MLPA subtelomere kit, and have described a robust analysis protocol that minimises both false positive and false negative results [6]. Nevertheless, confirmation of single probe abnormalities is prudent. In the data described here, all but 4 of the subtelomere abnormalities were confirmed, either by using a second MLPA subtelomere kit containing probes at nearby loci within the subtelomere, or by FISH, or by confirming the abnormality in another family member. The microdeletion MLPA kit contains several probes within each microdeletion region, and at least two probes within each region showed imbalance for six out of the seven abnormalities reported here. Nevertheless, where FISH probes were available, these were used to confirm the imbalance. Even when subtelomere results were confirmed, their clinical significance was not always clear. Where the same abnormality was found in a parent with apparently normal clinical phenotype, it is likely that the imbalance may represent polymorphic variation; it is interesting that all such abnormalities found in this group were duplications, perhaps reflecting population copy number variation such as that described recently [9-11]. De novo abnormalities, however, are considered very likely to be associated with the patients' developmental delay or dysmorphism.

In one patient (#18), originally assigned a normal karyotype, MLPA revealed imbalance which was visible on retrospective examination of the G-banded chromosomes. This underlines the difficulty of comparing prevalence of such abnormalities in publications from different centres, as prevalence of detection by MLPA (or other molecular cytogenetic methods) will be apparently greater in centres where G-banded chromosome analysis is carried out at lower quality, and will also depend on the selection criteria for the patient cohort. Further studies on these patients are in progress to assess the exact extent of the imbalance.

Of the submicroscopic abnormalities in the subtelomere group, one unbalanced translocation, 5 deletions and 11 duplications were detected, indicating that duplications may be more prevalent. Similarly, there were 3 deletions and 4 duplications in the microdeletion group; here, one might have predicted that those with deletions would be targeted for specific testing because of the characteristic syndromic features associated with such deletions, and therefore would not be included in the tested group. This would, however, depend on the diagnostic skill of the referring physician; the non-specific phenotypes reported here associated with known microdeletions (for instance in patients #12 and #13) underlines the variability in the presentation of these syndromes.

The copy number variation in the human genome reported recently [9-11] is thought to under represent smaller variants [12,13] and so it seems likely that testing with ~60 base pair probes will uncover further polymorphic variation. For instance, in the data presented here, patients #18, #21 and #25 all carry inherited duplications of 9p; this region may therefore be subject to population polymorphism of no clinical significance, and be an incidental finding in these cases. However, unless carrier parents' phenotypes are carefully assessed, an effect of variable penetrance cannot be completely excluded.

All the patients reported here were referred for Fragile X testing; none carried expansion mutations. At our Centre, Fragile X testing is carried out on average for 800–1000 patients per year, of whom < 1% are found to carry an expansion mutation. This is in contrast to the 7.2% detection rate for submicroscopic imbalance detected by MLPA, although as none of the subtelomere results were established as de novo, only the abnormalities found by microdeletion/duplication testing could be definitively established as of clinical significance.

Previous publications detailing submicroscopic imbalance detected by MLPA in patients with developmental delay/dysmorphism have reported abnormality detection rates of 5.3% [4], 5.9% [3], 6.7% [5] and 5.9% [6] when testing subtelomeric loci, 5.8% [7] when testing microdeletion loci, and 13.8% [8] when testing at both subtelomeric and microdeletion loci. Our overall detection rate by MLPA, for a larger cohort than these previous studies, was 7.2%; however, as discussed above, comparison between different centres is difficult because of the differences in resolution of G-banded chromosome analysis, and in the selection criteria for the patients.

## Conclusion

In conclusion, this paper reports the largest cohort to date of patients with developmental delay and/or dysmorphism, referred for the investigation of chromosome abnormality and tested for submicroscopic imbalance using MLPA. The importance of robust analysis protocols, confirmation of single probe findings, and appropriate family studies is emphasised. A comprehensive review [14] of available methodologies for assessing imbalance at subtelomeres concluded that MLPA is currently the method of choice, although the use of real-time PCR and microarrays may become more widespread as affordable commercial platforms become available [15]. Interestingly, a recent comparison of approaches to the investigation of patients with developmental delay [8] suggests that a strategy of replacing karyotyping with MLPA as a first test, followed by microarray analysis, may prove effective in terms of detection rate and cost-effectiveness. Of course, in the long term, it has been suggested that microarrays as a first test may prove to be the most efficient as the cost of this technology falls further [16]. In the UK, karyotype analysis as the "gold standard" and first test for children with developmental delay and/or dysmorphism is currently considered to be Best Professional Practice. The findings presented here suggest that the abnormality detection rate using a strategy of karyotype analysis followed by MLPA for subtelomeres and microdeletion loci will give a diagnostic yield considerably in excess of that gained by a strategy of karyotype analysis and Fragile X testing, and may be the best approach until microarray testing is generally validated as routine diagnostic tool, and becomes economical enough to replace karyotyping as the first test for these patients.

## Methods

### Karyotype analysis

Karyotype analysis was carried out on G-banded chromosomes prepared from peripheral lymphocytes by standard laboratory procedures. Preparations were generally analysed at quality 6 G-banding. Fragile X testing was performed with a biplex PCR to amplify the repeat regions at the FRAXA and FRAXE loci, using standard fluorescent PCR amplification. Products were then analysed using an ABI 3730 analyser (Applied Biosystems, USA) [17].

### MLPA analysis

MLPA analysis was carried out using kits (P036, P036B, P064, P064B and P069; MRC-Holland, The Netherlands), according to the manufacturer's protocols. Initial subtelomere testing used kit P069, which was followed by testing with kit P036 or P036B if an abnormality was detected. Analysis was according to our previous publication [6]. The MLPA microdeletion P064B kit contains probes for the following regions; 1p36 telomeric region, 7q11.23 Williams syndrome region, 17p11.2 Smith-Magenis region, 17p13.3 Miller-Dieker region, 22q11.21 DiGeorge region, 15q11.2 Prader-Willi/Angelman's region, 20p12.2 Alagille region, 7p21 Saethre-Chotzen syndrome, 5q35.3 Sotos syndrome region.

### Fluorescence in situ hybridization

Fluorescence in situ hybridization (FISH) confirmation used subtelomere and microdeletion probes from Abbott-Vysis (ToTelVysion; UK), Cytocell (Aquarius; UK) or MP Biomedicals (QBiogene; CA, USA), according to manufacturers' protocols. Subtelomere probes for confirmation were chosen according to the available information on genomic position relative to that of the MLPA probes.

## Competing interests

The author(s) declare that they have no competing interests.

## Authors' contributions

JWA developed the analysis protocol, carried out the MLPA testing and data analysis, and assisted in drafting the paper; KM and CO conceived the project and obtained the funding; KM analysed the data and assisted in drafting the paper; ZD provided critical input in the karyotype interpretation; CO analysed the data and wrote the paper.
